# Supplementary material for: (E)-2,6,10-Trimethyldodec-8-en-2-ol: An Undescribed Sesquiterpenoid from Copaiba Oil
Source: Molecules. 2021 Jul 23;26(15):4456. doi: 10.3390/molecules26154456 (PMC8348878; doi:10.3390/molecules26154456)
Supplement: Supplementary file 1 [file molecules-26-04456-s001.zip › molecules-1241643-supplementary.pdf]

Supplementary material

(*E*)-2,6,10-Trimethyldodec-8-en-2-ol: An Undescribed Sesquiterpenoid from Copaiba Oil

Mohammed F. Hawwal[1,3], Zulfiqar Ali[2,]*, Mei Wang[4], Jinping Zhao[2], Joseph Lee[2], Omer I. Fantoukh[3], and Ikhlas A. Khan[1,2,]*

[1] Department of BioMolecular Sciences, Division of Pharmacognosy, School of Pharmacy, The University of Mississippi, University, MS 38677, USA; mhawwal@go.olemiss.edu (M.F.H); ikhan@olemiss.edu (I.A.K.)
[2] National Center for Natural Products Research, School of Pharmacy, The University of Mississippi, University, MS 38677, USA; zulfiqar@olemiss.edu (Z.A); jianping@olemiss.edu (J.Z.); jclee1@olemiss.edu (J.L); ikhan@olemiss.edu (I.A.K.)
[3] Department of Pharmacognosy, College of Pharmacy, King Saud University, Riyadh 4545, Saudi Arabia; mhawwal@ksu.edu.sa (M.F.H); ofantoukh@ksu.edu.sa (O.I.F.)
[4] Natural Products Utilization Research Unit, Agricultural Research Service, U.S. Department of Agriculture, University, MS 38677, USA; meiwang@olemiss.edu (M.W.)

*Correspondence: zulfiqar@olemiss.edu (Z.A.); ikhan@olemiss.edu (I.A.K.); Tel.: +1-(662)9151090 (Z.A. & I.A.K.)

Abstract: The use of copaiba oil has been reported since the 16th century in Amazon traditional medicine, especially as an anti-inflammatory ingredient and for wound healing. The use of copaiba oil continues today, and it is sold in various parts of the world, including the United States. Copaiba oil contains mainly sesquiterpenes/, bioactive compounds that are popular for their positive effect on human health. As part of our ongoing research endeavors to identify the chemical constituents of broadly consumed herbal supplements or their adulterants, copaiba oil was investigated. In this regard, copaiba oil was subjected to repeated silica gel column chromatography to purify the compounds. As a result, one new and seven known sesquiterpenes/sesquiterpenoids were isolated and identified from the copaiba oil. The new compound was elucidated as (*E*)-2,6,10-trimethyldodec-8-en-2-ol. Structure elucidation was achieved by 1D- and 2D NMR and GC/Q-ToF mass spectral data analyses. The isolated chemical constituents in this study could be used as chemical markers to evaluate the safety or quality of copaiba oil.

Keywords: copaiba oil; *Copaifera*; Fabaceae; new sesquiterpenoid; sesquiterpenes; NMR

Contents:

Figure 1S. GC/MS total ion chromatogram of investigated copaiba oil.

Figure 2S. ^{1}H NMR spectrum of **1** in $CDCl_3$ at 500 MHz.

Figure 3S. ^{13}C NMR spectrum of **1** in $CDCl_3$ at 125 MHz.

Figure 4S. COSY spectrum of **1** in $CDCl_3$.

Figure 5S. HSQC spectrum of **1** in $CDCl_3$.

Figure 6S. HMBC spectrum of **1** in $CDCl_3$.

Figure 7S. IR spectrum of **1**.

Figure 8S. GC/Q-ToF mass spectrum of **1**.

Figure 9S. Tentative mass fragmentation associated with **1**.

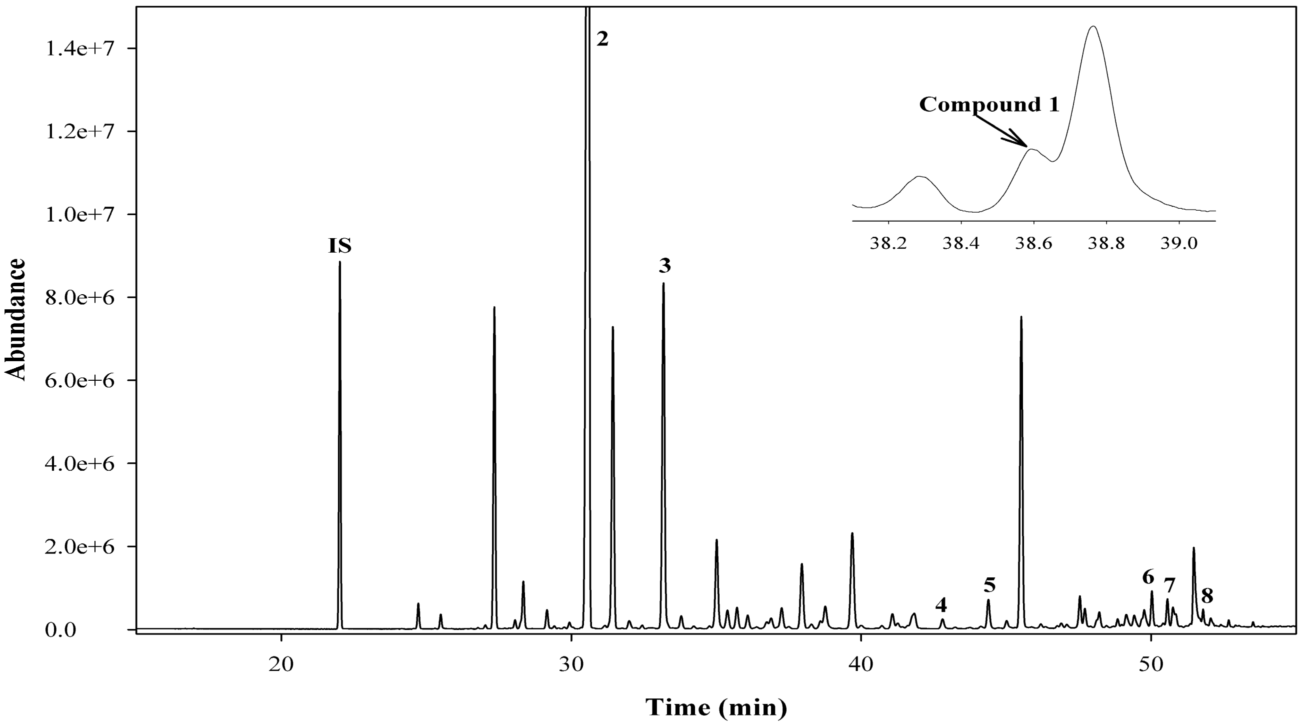

Figure 1S. GC/MS total ion chromatogram of investigated copaiba oil.

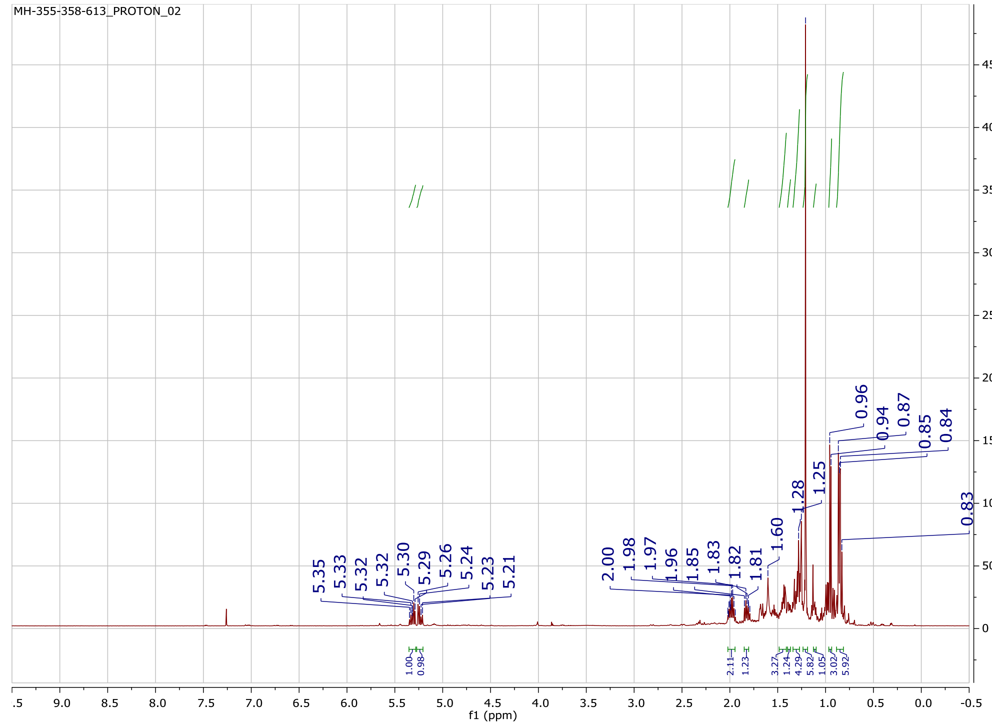

Figure 2S. ^{1}H NMR spectrum of **1** in $CDCl_3$ at 500 MHz.

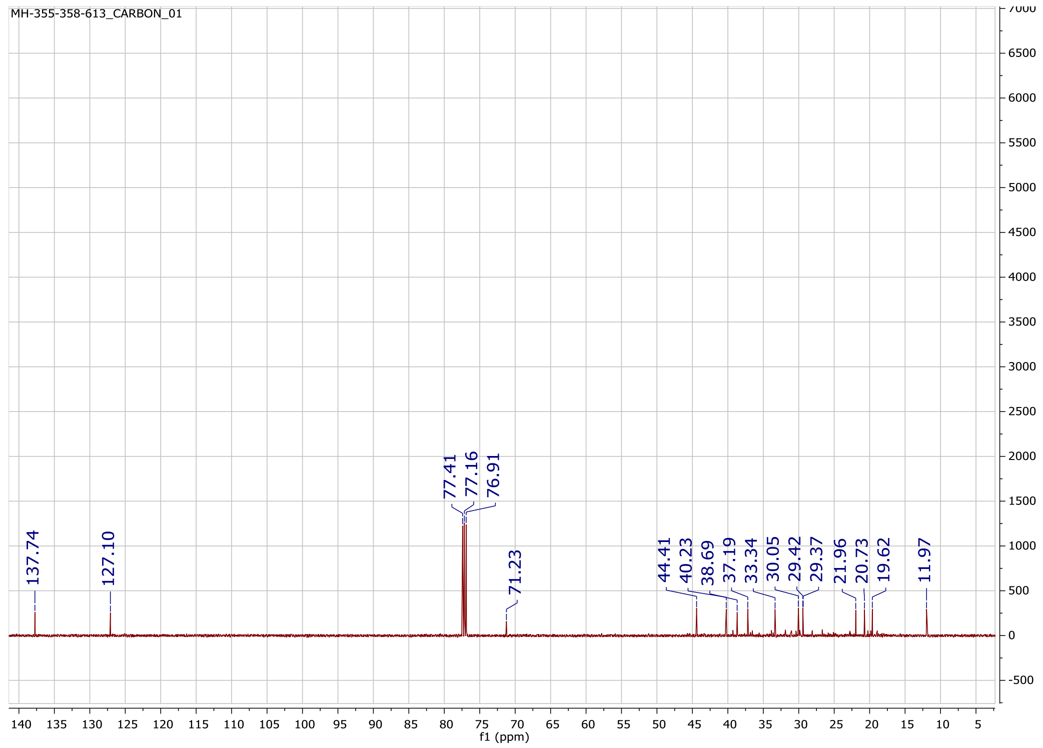

Figure 3S. ^{13}C NMR spectrum of **1** in $CDCl_3$ at 125 MHz.

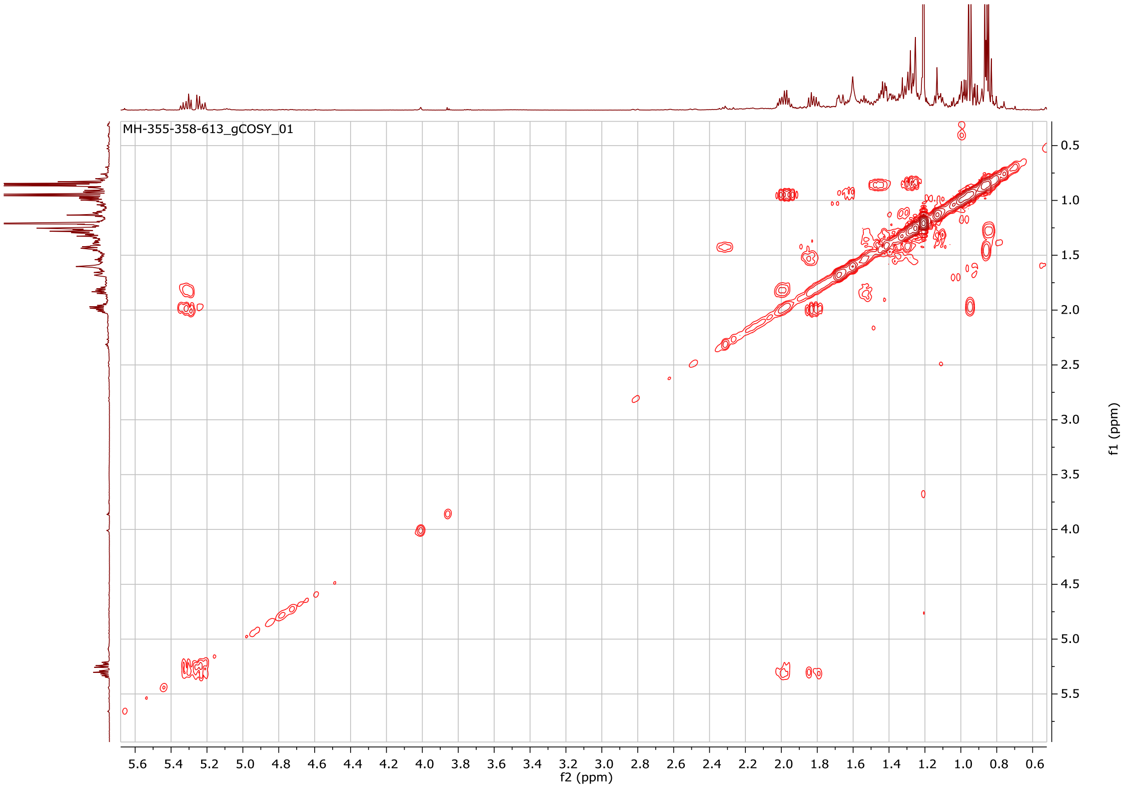

Figure 4S. COSY spectrum of **1** in $CDCl_3$.

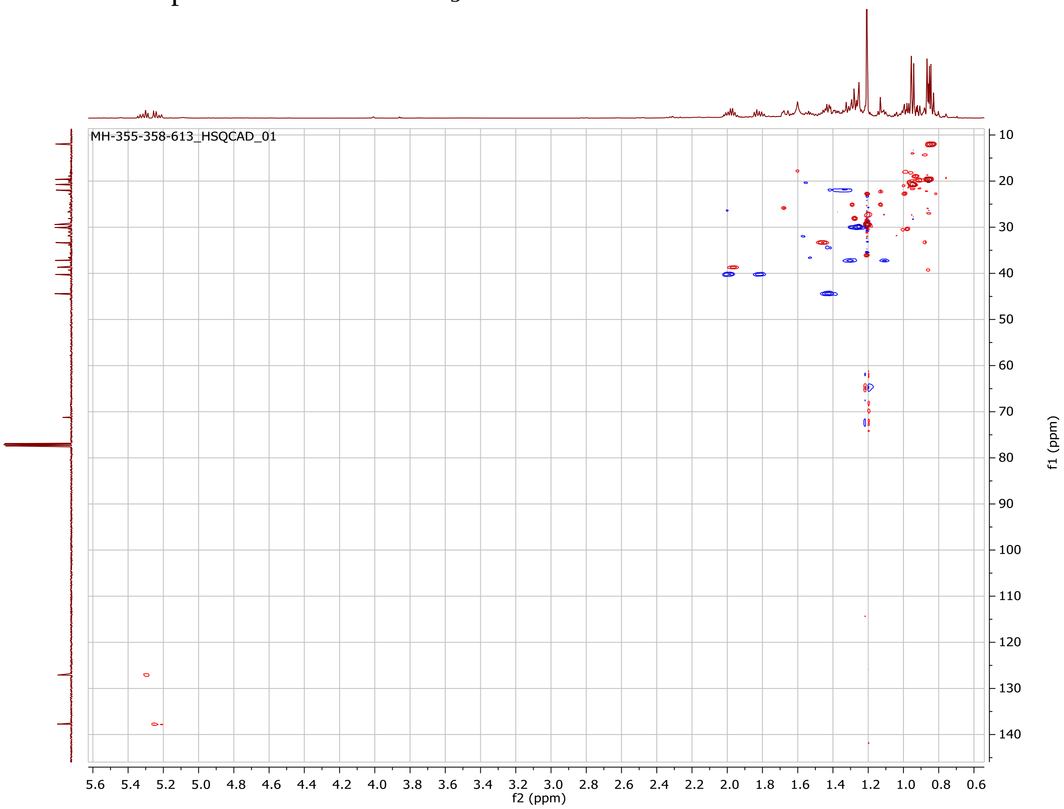

Figure 5S. HSQC spectrum of **1** in $CDCl_3$.

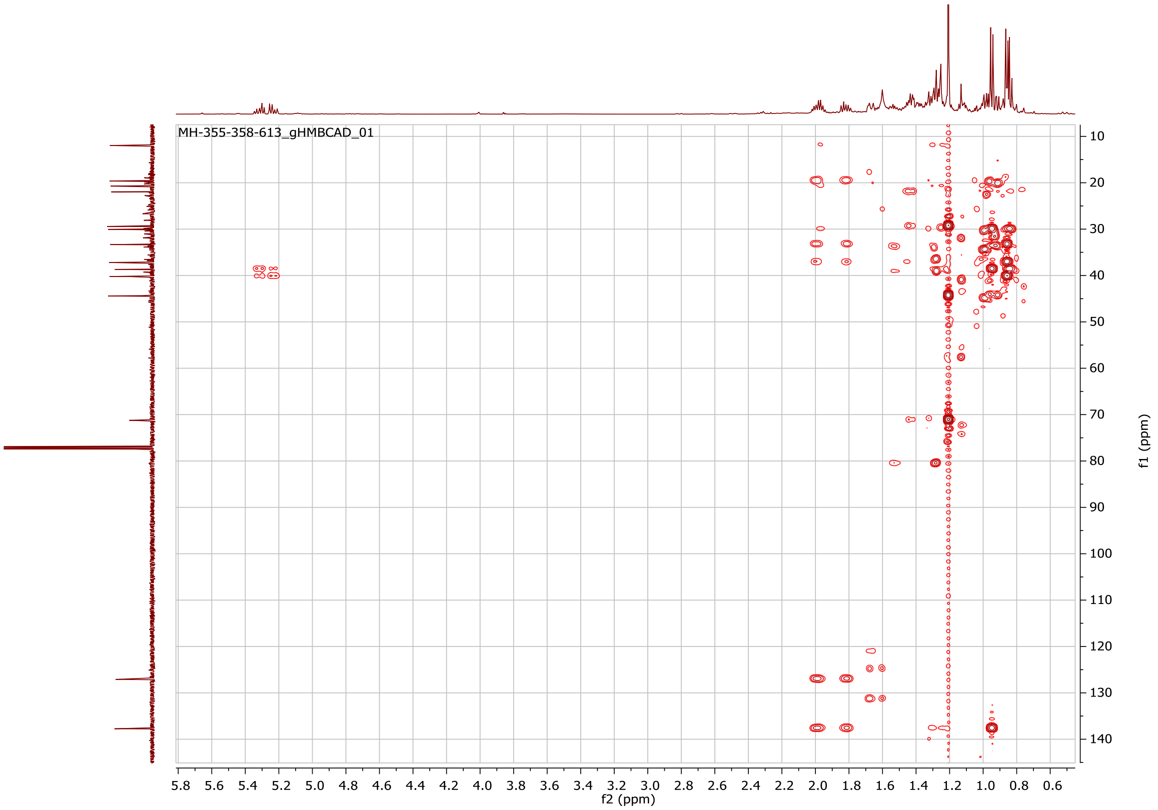

Figure 6S. HMBC spectrum of **1** in $CDCl_3$.

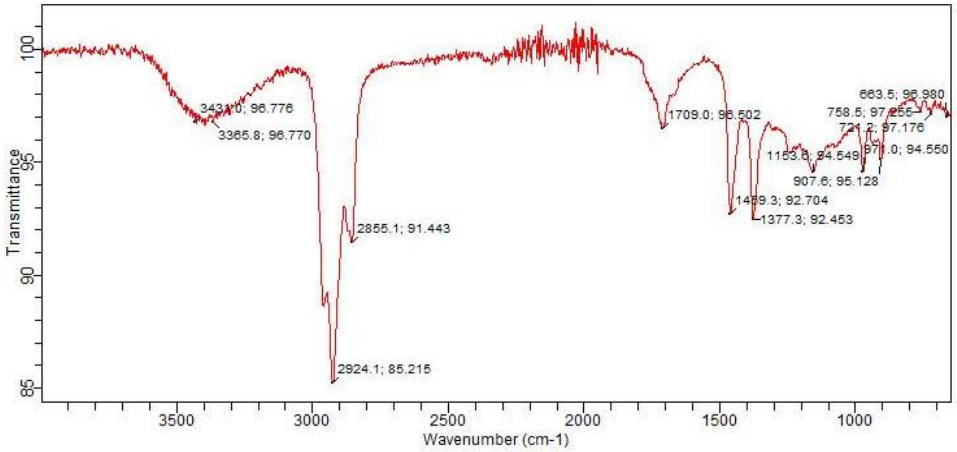

Figure 7S. IR spectrum of **1**.

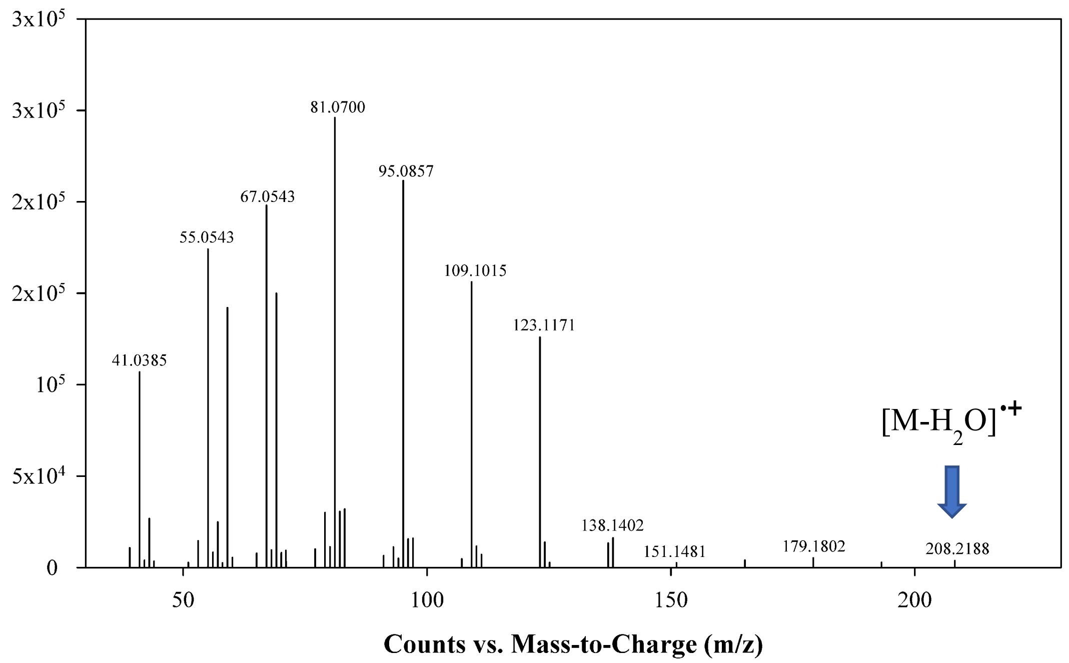

Figure 8S. GC/Q-ToF mass spectrum of **1**.

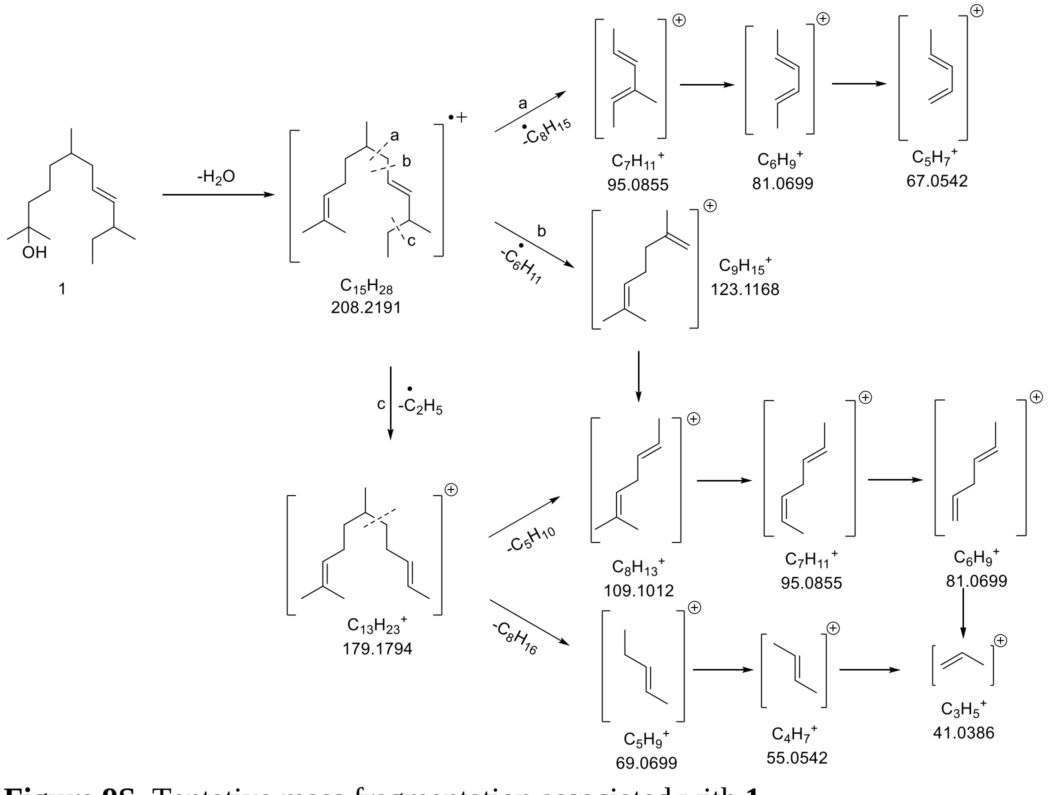

Figure 9S. Tentative mass fragmentation associated with **1**.
